# Promising Applications of Tumor Spheroids and Organoids for Personalized Medicine

**DOI:** 10.3390/cancers12102727

**Published:** 2020-09-23

**Authors:** Zarema Gilazieva, Aleksei Ponomarev, Catrin Rutland, Albert Rizvanov, Valeriya Solovyeva

**Affiliations:** 1 Institute of Fundamental Medicine and Biology, Kazan Federal University, 420008 Kazan, Russia; ZarEGilazieva@kpfu.ru (Z.G.); AlesPonomarev@stud.kpfu.ru (A.P.); Albert.Rizvanov@kpfu.ru (A.R.); 2 Faculty of Medicine and Health Sciences, University of Nottingham, Nottingham NG7 2UH, UK; Catrin.rutland@nottingham.ac.uk

**Keywords:** 3D tumor models, spheroids, organoids, personalized medicine, oncological diseases, drug screening, biobank

## Abstract

**Simple Summary:**

Presently, the investigation of the tumor is carried out using different models. One of the promising areas is the creation of 3D tumor models, such as spheroids and organoids. These models are close in properties and organization to a native tumor. This review includes information about 3D tumor models, their differences, and methods for creating spheroids and organoids. The technical aspects of these models are summarized in this review. It provides an overview of the main uses of these models in personalized medicine to create a promising screening model for therapeutic agents and describes the latest research in this area. The combination of 3D tumor models and high-throughput approaches of personalized medicine (transcriptome, genomic, metabolomic, etc.) will open up new possibilities for the creation of improved therapy for oncological diseases.

**Abstract:**

One of the promising directions in personalized medicine is the use of three-dimensional (3D) tumor models such as spheroids and organoids. Spheroids and organoids are three-dimensional cultures of tumor cells that can be obtained from patient tissue and, using high-throughput personalized medicine methods, provide a suitable therapy for that patient. These 3D models can be obtained from most types of tumors, which provides opportunities for the creation of biobanks with appropriate patient materials that can be used to screen drugs and facilitate the development of therapeutic agents. It should be noted that the use of spheroids and organoids would expand the understanding of tumor biology and its microenvironment, help develop new in vitro platforms for drug testing and create new therapeutic strategies. In this review, we discuss 3D tumor spheroid and organoid models, their advantages and disadvantages, and evaluate their promising use in personalized medicine.

## 1. Introduction

Presently, more than 100 types of tumors have been identified [1,2]. These tumors have different localizations within tissues and consist of a variety of tumor cell types. However, even one type of malignant tumor can have differing expression patterns of tumor-associated markers, the presence of which depends on the localization of the tumor, the stage of the disease, and various genetic factors [1,2]. For example, a colorectal or breast tumor can have different molecular subtypes [3]. Due to the heterogeneity of malignant tumors, traditional methods of treatment (chemotherapy and irradiation) have low efficiencies or show positive effects in only some groups of patients. Thus, it is necessary to take into account the individual characteristics of the patient in order to prescribe an effective treatment [2].

The development of personalized medicine includes methods of preventing, diagnosing and treating a pathological condition based on the patient’s individual characteristics [4]. Such individual characteristics consist of genetic, epigenetic, transcriptomic, proteomic, and metabolomic markers of the patient’s entire body, tissues and cells [5]. In addition, it is possible to investigate gene expression profiles and recognize changes that are associated with oncological diseases [2].

Personalized medicine obtains information about the disease of a specific patient to select a drug and the correct dosage, as well as optimizing the treatment procedure [6]. Personalized medicine also has influences on drug preclinical studies, determining not only effectiveness and safety, but also reducing the cost of pharmaceutical development [4].

The effects of potential drugs on whole organs/tissues are usually investigated in in vivo models. These models have drawbacks associated with the possible effects of other systems on the processes in a specific organ or tissue. Therefore, the development of screening in vitro models has implications and holds potential for therapeutic drug research. An effective screening system should be as close as possible in properties and organization to a native tumor. Monolayer cell cultures are still the main in vitro model [7,8,9]. However, this model is quite different in comparison to native tumors. Significant limitations are present in relation to the study of the immune system, microenvironment, and stromal compartments effect on tumor cells. Oxygen gradients, growth factors, metabolites and the presence of necrotic, hypoxic, resting and proliferating cells are present in a native tumor and limited in monolayer cell cultures [10]. In addition, following long-term cultivation of tumor cell lines, the genetic heterogeneity of the original tumor is excluded [11]. Research can also be carried out on tumor xenografts, but it is expensive and quite a time-consuming procedure [12]. The solution to this problem is 3D tumor model development, which would accurately mimic the features of the tumor and tumor microenvironment (TME).

The use of 3D tumor models in research could eliminate the limitations that exist in 2D model systems based on immortalized cells or cell lines of the tumor origin. Research in 3D tumor models is more consistent with in vivo studies because it has important properties that make it more like native tissue. These models can contain several types of cells. The tumor architecture, genetic heterogeneity and interaction of the tumor cells and stroma are also preserved [9]. One of the features of the tumor is intra-tumoral stiffness. For example, increasing the stiffness of breast tumors leads to poor prognosis. With this characteristic, 3D tumor models can be used to study the change in stiffness during metastatic invasion and cytoskeleton reprogramming [13].

According to the literature, cancer stem cells (CSCs) play a significant role in the progression of oncology. CSCs are a small population of cells capable of self-renewal, cloning, growth, and metastasis [14]. In addition, CSCs are capable of conferring resistance to therapeutic drugs. The effect of tumor stem cells on the disease development and identifying new specific agents that target the CSC signaling pathways can be accurately investigated using a 3D model system [15]. Using 3D models can enable investigations relating to intercellular contacts, TME and the interaction of immune and tumor cells. Additionally, mathematical modeling can to be applied in 3D model research [16].

It is important that 3D models can be obtained from each patient and provide the appropriate therapy for this patient, taking into account all their characteristics, and make a significant contribution to the understanding cancer biology in general but also specifically for the individual.

## 2. Tumor Spheroids and Organoids

There are a number of technologies used to obtain 3D tumor models, which are developed and take into account the characteristics of a native tumor [17]. Spheroids and organoids can be distinguished among them [18]. The 3D tumor spheroids are self-organizing cultures of tumor cells with a rounded morphology and predominant intercellular interactions [19,20]. Spheroids can proliferate and grow as free-floating structures [21]. Tumor spheroids larger than 500 µm in diameter are often characterized by hypoxic areas and necrotic centers and the formation of zones occurs due to the lack of nutrients and limited oxygen transport [22]. A feature of the spheroid inner part is an acidified microenvironment (pH range 6.5–7.2) [23]. In response to the low pH level of the environment, cells in the senescent and necrotic zones begin to produce factors that enhance the proliferation of malignant cells and their survival [24].

There are several types of tumor spheroids or spheres. The most frequently used spherical models include: (I) a multicellular spheroid which was obtained by culturing tumor cell lines on non-adhesive culture dishes; (II) an oncosphere, which is a model of expansion of cancer stem cells (CSCs) in a serum-free medium supplemented with growth factors; and (III) an organotypic multicellular spheroid obtained by mechanical and enzymatic dissociation of tumor tissue [17].

Currently, several terms are used to describe spheroids. The terms “sphere”, “tumor sphere” [25] and “oncosphere” [26] are used to describe the spheres obtained from CSCs. Tumor spheres are a mixture of CSCs and tumor cells, since CSCs are known for their ability to proliferate and differentiate [27]. This model is commonly used to evaluate and characterize CSCs in vitro [28]. Enrichment with tumor stem cells gives certain properties to spheres, the study of which is of clinical importance. The name of the spheres can arise from whichever tissue the spheroid is derived from. For example, the spheres of the brain and mammary gland stem cells were called “neurospheres” [29] and “mammospheres” [30], respectively. Spheres from the colon cancer cell line were called “colonospheres” [31]. Later, the spheres were called “spheroids” [32].

Organotypic multicellular spheroids are required for accurate reproduction of TME. Tumor cells in these spheroids are surrounded by non-tumor cells and stromal components that are commonly found in TME [28]. There are several methods for obtaining organotypic tumor spheroids, including enzymatic dissociation of tissues to isolate spheroids with cellular heterogeneity, similar to the primary tumor [33]. In many cases, these cultures are created using mechanical dissociation of tumor tissues. As a result, this model is capable of proliferation and can retain many of the histological features within the original tissues [28,34].

The creation of multicellular tumor spheroids usually occurs by culturing tumor cell lines under normal conditions similar to 2D cultures. Therefore, multicellular tumor spheroids can be methodologically considered as an extension of standard 2D culture of tumor cell lines [28]. The main difference from 2D cultures is that cells in this model are grown in the form of spheres in suspension cultures or in other conditions that promote intercellular adhesion. Unlike other spheroids, multicellular tumor spheroids have a lower histological similarity when compared to native tissue. However, these spheroids maintain the metabolic and proliferative properties of the primary tumor and may exhibit chemoresistance [35]. The advantages of this model, compared to other 3D systems, are cell clonality, ease of maintenance and genetic manipulation. This makes spheroids a suitable tool for high-throughput drug screening [36].

All listed models are used in studies of chemoresistance, radioresistance, oncogenicity, invasion and migration. Despite the fact that spheroids have a common three-dimensional conformation, various methods of their preparation allow for creation of spheroids with additional properties. Thus, selection of the most appropriate spherical tumor model should be carried out based upon the purpose of the investigation and the type of tumor.

Organoids are also a type of 3D cell culture. This term is usually used for a three-dimensional culture of normal cells and tissues [37,38]. They are aggregates of different cell types, often used in combination with a biocompatible three-dimensional scaffold. Organoids are capable of self-renewal, self-organization and the manifestation of the functionality of the organ that it imitates. Organoids maintain a cellular composition and structure similar to the tissue from which they originate. When organoids are generated, various cell types occupy their own niches, which ensures that their interactions, and physiological similarities are preserved. These cell equivalents can be cryopreserved and multiplied [39]. Organoids can be obtained from embryonic or induced pluripotent stem cells [40]. Organoid cultures derived from adult stem cells were created from leucine-rich repeat-containing G-protein coupled receptor 5 (Lgr5)-expressing mouse intestinal stem cells [41].

Similarly, this term is used for 3D tumor cell structures. Whitehead et al. spontaneously cultivated LIM1863 colon carcinoma cell line as organoids [42,43]. To date, organoids of tumors have been obtained from various organs, including the colon [44,45], pancreas [46], breast [47], stomach [48], lung [49], esophagus [50], urinary bladder [51], kidney [52] and liver [53]. It should be noted that one of the important features of tumor organoids is that they are genetically and phenotypically more similar to a native tumor, forming intratumoral heterogeneity [45,47,48]. In conclusion, 3D tumor models of spheroids and organoids can become transitional links between in vitro and in vivo studies and play vital roles in testing new pharmaceutical compounds.

## 3. Methods for Obtaining Spheroids and Organoids

The 3D cultures have specific properties and, therefore, their cultivation requires conditions that differ from 2D cultures (Table 1) [54]. There are a number of methods for obtaining tumor spheroids. These methods are primarily based on the prevention of cell adhesion to the culture plastic surface. Not all primary tumor cells and cell lines are capable of forming spheroids. Some tumor cells form spheres spontaneously, whilst others require additional manipulations [36].

The hanging drop method involves the cells being placed in a drop of culture medium and incubated under standard conditions until spheroids are formed [55]. This method does not require special equipment and can be used to co-culture cell lines. Formation of the spheroids usually occurs within 24 h, but it may take longer [56]. It has been shown that the addition of 25% methocel in medium drop enhances the formation of spheroids. The methocel used for these experiments was prepared by dissolving 6 g of carboxymethyl cellulose in 500 mL of medium. Methocel prevents cell clumping and is inert whilst maintaining viscosity. This addition makes it possible to successfully generate more uniform spheroids and requires less cultivation time [54,55]. The hanging drop method is a simple, low cost, high performance method. However, the disadvantages of the method include difficulties manipulating and evaluating various therapeutic drugs [57]. Moreover, it is possible to create the combination of the hanging drop technique and the polydimethylsiloxane platform. It has been shown that collagen fibrils improve the formation of spheroids even from a small number of cells (about 200 cells per spheroid). In addition, the authors showed that this platform could be used to study the biology of tumor cells [55].

There are dynamic methods for generating spheroids based on the use of rotating flasks, shakers and bioreactors. Spontaneous cell aggregation occurs due to constant agitation. It is a simple and highly productive method for controlling nutrient metabolism, but it requires specialized equipment that is not always present in laboratories but is easily available. This method does not enable the cultivation of individual spheroids [58]. The liquid overlay technique involves the formation of spheroids using non-adhesive surfaces [59,60]. There are different variations of this method depending on the type of dish in which the cells are cultured. For example, cells can be pelleted in the cone of a well by smooth centrifugation using V-shaped [59] or U-shaped wells [61,62]. Multi-well plates can be used to create spheroids that are more homogeneous. Scherer et al. grew spheroids from C8161 and MDA 231 cells in 96 well round bottom plates [63]. It has been shown that in 96 well plates, optical coherence tomography can be used for 3D imaging [64]. In addition, hanging drop multi-well microtiter plates have been used by Amann et al. as a method of cultivating 3D mono and co-cultures of A549 and Colo699 [65].

Various non-adhesive substrates, such as agarose [36] and poly-HEMA [61], prevent cell adhesion and alter the morphology of spheroids. This simple, low-cost method does not require specialized equipment, and offers the possibility of cultivating single spheroids. Optimization of protocols in order to obtain spheroids of uniform size and shape is a disadvantage. Additionally, the process of preparing non-adhesive dishes can be laborious [66]. Ivascu and Kubbies presented the process of single homogeneous spheroid cultivation of twenty tumor cell lines using 5 × 10^3^ tumor cells and 96-well conical-bottomed plates to create spheroids. Centrifugation of the plate for 10 min at 1000× *g* led to the deposition of cells at the bottom of the well, and the addition of a small amount of Matrigel to the culture medium improved the formation of spheroids [61].

**Table 1:** Comparative characteristics of 3D tumor models.

3D Models	Materials for 3D Models Preparation	Advantages	Disadvantages
Spheroids [19,29,55,67,68,69,70,71]	(1) Various plates and rotors can be used for cultivation (spinner flasks, rotary cell culture systems, poly-2-hydroxyethyl methacrylate (poly-Hema)-coated plates, liquid overlay, micropatterned plates, low binding plates, microfluidics device). (2) The addition of B27, epidermal growth factor (EGF) and fibroblast growth factors (FGFs) are essential for spheres obtained from CSCs. Spheroids can be cultivated, including fetal bovine serum (FBS) and without it.	(1) Possibility of: (a) Creation of co-culture. (b) Culturing cells without special equipment. (c) Cultures without expensive cultivation methods. (2) Gas, nutrient and pH gradients are present.	(1) The difficulty of forming homogeneous spheroids. (2) Fragile spheroid structure. (3) Gradient structure complicates drug testing.
Organoids [44,72,73,74,75]	(1) Cells and cell aggregates culturing are cultured on various matrices (Matrigel, collagen type I, HA (hyaluronic acid) hydrogel, PEG hydrogel, Fibrin/laminin hydrogel). (2) Culture supplements depending on the tissue type are essential. The main ones include: Wnt, nicotinamid, N-acetylcysteine, R-spondin-1, FGFs, noggin and molecule inhibitors (Y27632, A-83-01, SB202190).	(1) Possibility of: (a) Creation of co-culture. (b) Reproduction of intercellular interactions and cell-ECM interactions. (c) Primary tumor cells long-term cultivation. (2) Stable at passaging.	(1) Expensive method. (2) The gradient of gases, nutrients and pH is not always reproducible. (3) Therapeutic responses may depend on the matrix.

It has been shown that microfluidics can also play a role in the formation of spheroids. Microfluidics include microchannels and microchambers in which circulating cells accumulate in chambers, thereby forming spheroids. Using this method, the size, shape and nutrient metabolism of spheroids can be controlled. The time taken to form spheroids in microchambers is relatively short; however, specialized equipment is required, and the process of extracting the spheroids is difficult [76].

Thus, protocol optimization for spheroid formation exists and there is also a need to eliminate existing limitations. One limitation is that the tested cell lines differ in their ability to form spheroids. Standardization for further research is further complicated by long-term cultivation, as well as the formation of spheroids heterogeneous in size and shape [55,61].

Cells and a matrix are needed to create organoids. The matrix can be of natural or synthetic origin. The matrix plays the role of structural support of cells in tissues and it is an important component of the cellular microenvironment, which includes the regulation of intercellular interaction [77]. Matrigel is widely used as a matrix because it has similarities to the extracellular matrix (ECM). It is characterized by a multicomponent protein fraction and growth factors obtained using the Engelbreth–Holm–Swarm murine sarcoma cell line [78]. In addition, natural matrices are used for the cultivation of organoids, such as hydrogels based on human liver fibrin and ECM [79,80].

Recently, synthetic matrices can be used to create organoids. The advantages of synthetic matrices are their purity (they do not contain growth factors and other proteins) and ease of production. An example is polyethylene glycol (PEG) matrices [81]. To reduce the rigidity of synthetic matrices, hybrid matrices can be created. Gjorevski et al. mixed a stable polymer backbone (sPEG, mechanically static PEG) with a degradable polymer (dPEG, mechanically dynamic PEG), and added the RGD peptide (Arg-Gly-Asp) and laminin-111. Changes in matrix rigidity improved the formation of organoids [82]. Moreover, it is possible to use a photo-crosslinkable macroporous hydrogel. This hydrogel is a cellulose spongy system composed of hydroxypropyl cellulose with the inclusion of methacrylic groups [83].

Thus, the inclusion of matrix components in in vitro systems allows 3D models to be obtained imitating the TME. Moreover, the availability of various designer matrices and their replaceable components is leading to increased experimental possibilities for organoid culture systems.

Tumor organoids can be obtained from tumor cells isolated from tumor tissue [84]. The first step in obtaining organoids from tumor tissue is mechanical or enzymatic degradation. Usually, enzymes such as collagenase IV, hyaluronidase, and trypsin-ethylenediamine tetra acetic acid are used for enzymatic degradation. After that, individual cells or cell clusters are mixed with a matrix and cultured in medium with growth factors, depending on the type of tissue [72,85]. The obtained organoids can be passaged and frozen. The use of ROCK inhibitors during the passage of organoids retards aging and supports cell proliferation [86]. Presumably, tumor organoids can also be obtained from normal organoids using gene-editing techniques [87]. For example, intestinal organoids from surgically resected intestinal tissues have been transformed into tumor organoids using CRISPR-Cas9 genome editing [88]. Such modeling of disease onset can provide insight into the initiation of cancer, its progression, impaired therapeutic response, and the discovery of new oncogenic proteins or antigens that can be targeted to create therapies [89]. However, the entire population of target cells may not contain mutations. The result of this creation is a clonal cell line with mixed genotypes in mutant 3D models. Nie et al. described this in a study where even after puromycin selection or FACS sorting, the cell line remains uneven. In addition, genetic insertions may not completely knock out a gene and disrupt its function [90]. Thus, this makes analysis difficult.

The literature describes a large number of tumors from which organoids can be initiated [91]. Weeber et al. successfully cultured organoids from metastatic colorectal carcinoma tissue [92]. Genetic analysis using SOLiD sequencing for 1977 genes showed that organoids are similar to metastases and also have common somatic mutations [92]. Organoids were also obtained from cervical carcinoma biopsy tissue. Genomic analysis showed that the organoids contained several mutations, two of which were common with the tumor tissue, including nonsynonymous mutation in MLH1 and a synonymous mutation in TFE3 [93]. Organoid cultures of hepatocellular carcinoma and cholangiocarcinoma retained the histological architecture, gene expression and genomic landscape of the original tumor [91]. During the cultivation of these organoids, the authors faced the problem of culture contamination with non-tumor organoids. A change in the protocol, which included an increase in the time of tissue digestion and the addition of dexamethasone and an inhibitor of Rho kinase, subsequently avoided this contamination [91]. Organoids from tumors of the gastrointestinal tract were glandular structures that formed a single lumen lined with a single layer of epithelial cells [94]. To study the phenotypic effects of CDH1 and RHOA mutations in organoids, the CRISPR-Cas9 technique was used [94]. Organoids obtained from the patient’s ovarian cancer tissue have similarities to native tissue and reproduce the cellular and molecular phenotype of the disease [95,96]. Interestingly, the addition of NRG1 significantly increased the number of organoids [95]. Puca et al. have showed genomic, transcriptome and epigenome similarities between organoids and biopsy of a prostate neuroendocrine tumor [86]. The organoids obtained from the patient’s metastatic tissue of castration-resistant neuroendocrine prostate cancer were infected with short hairpin RNA targeting EZH2. The knockdown of the gene reduced the properties of stem cells and the function of tumor cells. In addition, these organoids have been used as a platform for drug screening using an EZH2 inhibitor [86]. Histological investigation of lung tumor organoids revealed pseudostratified airway epithelium containing basal, secretory and multicilicated cells. It was confirmed that the secretory cells as well as cilia were functional [49]. Hubert et al. described the possibility of obtaining and cultivating glioblastoma organoids [97]. These organoids contained rapidly dividing SOX2^+^, OLIG2^+^ and TLX^+^ stem cells. Furthermore, orthotopic transplantation of the obtained organoids led to the development of a tumor [97]. Interestingly, tumor organoids can have differing morphologies, despite the fact that they were obtained from the same tissue type. Kenny et al. showed that tumor organoids obtained from 25 different breast cancer cell lines had different shapes (round, massive, grape-like and stellate) after four days of cultivation [98]. Similar results were obtained with prostate tumor cell lines [99]. Presumably, such morphological diversity may arise from differences in the genetic profile or external factors such as different levels of oxygenation or ECM composition.

An air–liquid interface can also be used for the cultivation of organoids. The air–liquid interface was used first to create gastrointestinal organoids [100], and then to create tumor organoids [101]. This system promotes long-term cultivation and improved cell proliferation.

Despite the fact that the use of organoids represents a promising new model for studying tumors, their cultivation is a long and expensive process. Some issues remain unresolved, which include tissue biopsy processing, environment optimization and matrix selection to create the same optimal conditions for the tumor organoids’ cultivation.

Currently, there is growing interest in the use of induced pluripotent stem cells (iPSCs) for basic research and drug improvement. iPSCs are reprogrammed somatic cells that can be supported indefinitely and directed towards differentiation into cells of any type [102]. Tumor-derived iPSCs can aid in cancer stem cell research. iPSCs can be used to observe associations between genotype and responses to anticancer drugs. Their wide developmental potential makes it possible to study the effects of a specific cancer genotype or specific driver mutations [103]. iPSCs were obtained from patients with familial cancer syndromes. For example, iPSCs have demonstrated carcinogenic potential and gene signatures for osteosarcoma, which usually develops in patients with Li-Fraumeni syndrome [104].

The main advantage of 3D models is the ability to reproduce more natural conditions of cell growth and interaction, and the use of iPSCs allows us to strengthen this system, increasing the complexity of the model, allowing the phenotype of the disease at the organoid level to be attributed to a specific molecular lesion and testing various therapies with the most approximate pathophysiology of the disease [105]. It has been shown that 3D structures derived from c-met-mutated iPSCs have molecular similarities to tissues of a patient with type 1 papillary renal cell carcinoma. Factors such as BHLHE40 and KDM4C were expressed in c-met-mutated kidney 3D models and the same type of tumor. It was also shown that these structures could be used for drug testing. The effects of sunitinib and cisplatin were studied on them [106].

Thus, combining these cells and 3D models or obtaining organoids from iPSCs can provide an improved model for predicting disease and tailoring personalized therapy for patients. In addition, these cells, presumably, will contribute to the long-term cultivation of organoids and the initiation of their formation through the inclusion of iPSCs.

## 4. Spheroids and Organoids for Personalized Medicine

**Figure 1:**
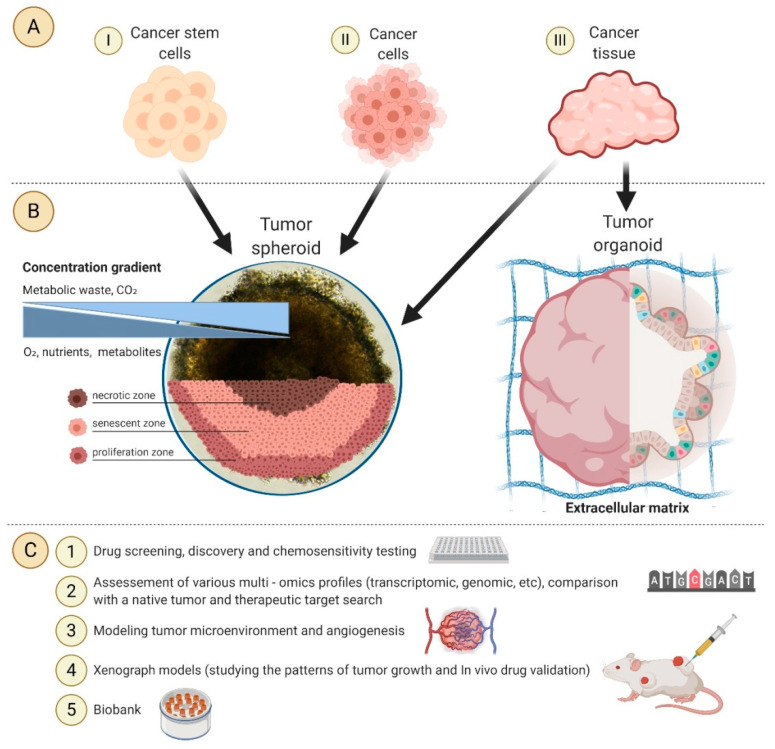
The application of 3D tumor models in personalized medicine: (**A**) Starting materials for tumor spheroids and organoids cultivation; (**B**) The structure of the tumor spheroid; (**C**) Application of tumor spheroids and organoids.

Currently, 3D models are widely used. It is possible to study tumor biology, intercellular interactions, microenvironment and genetic profile using 3D tumor models (Figure 1). It should be emphasized that these 3D tumor models play a huge role in the development of anticancer drugs and can contribute to the identification of new-targeted cancer therapies. It has been shown that spheroids of some cell lines can exhibit resistance to cytotoxic drugs such as taxol, cisplatin, 5-fluorouracil and doxorubicin [107,108]. Thus, the spheroids of the MDA-MB-157 cell line exhibit complete resistance to paclitaxel [109]. It can be assumed that the main factor that contributes to drug resistance is insufficient penetration and distribution of drugs in the spheroid cell mass. Therefore, for screening antitumor agents on spheroids, it is necessary to take into account their gradient structure, including the presence of hypoxia and cell proliferation areas [36]. For example, absorption of irinotecan into HCT-116 colon cancer spheroids revealed accumulation of the active metabolite SN-38 in the outer layers of the aggregate, whereas irinotecan was concentrated in the necrotic core of spheroids [110].

Hypoxia can induce changes in tumor cells and lead to altered gene expression; for example, the hypoxia inducible factor (HIF) 1 [36]. It has been shown that inhibition of HIF-1α in breast tumor spheroids induces doxorubicin accumulation, thereby reducing drug resistance [111]. Moreover, this addition increased activity of caspase-9, which plays a significant role in cell apoptosis. In addition, tumor spheroids can be used to evaluate the drug delivery system. For example, Pattni et al. developed a delivery system for the NCL-240 proapoptotic drug; the efficacy was then evaluated on spheroids from NCI/ADR-RES ovarian cell line [112].

Drug testing can be performed on spheroids formed from tumor tissue. For example, Halfter et al. compared the effects of therapeutic agents on spheroids obtained from tissue and a breast tumor cell line, which showed the difference between these groups [113]. Jeppesen et al. held chemosensitivity screening using spheroids of primary colorectal adenocarcinoma cells from five patients, which demonstrated individual response profiles [114]. Spheroids retained differences between patients in sensitivity to drugs and combinations commonly used to treat colorectal cancer [114]. Thus, tumor spheroids can play a significant role in drug testing before their clinical application because their cultivation is quite simple and inexpensive, and they have some properties that characterize a tumor.

Tumor organoids have been used to screen for therapeutic drugs such as 5-fluorouracil, oxaliplatin, irinotecan, doxorubicin and docetaxel [115]. The toxicity of cisplatin has been successfully assessed using human renal carcinoma organoids [116]. The distribution of drugs can be examined using matrix-assisted laser desorption/ionization mass spectrometry imaging (MALDI-MSI). For example, Liu et al. assessed the distribution of irinotecan and its metabolites in patient-derived colorectal tumor organoids [117]. In addition, it was found that ERK signaling pathway might be a potential therapeutic target for patients with primary liver tumors [91]. Broutier et al. used primary liver cancer organoids to demonstrate the correlation between some drug susceptibility and mutation profiles, and tested SCH772984 (ERK inhibitor) as a potential novel therapeutic agent [91]. Frappart et al. created a predictive platform for therapeutic agents based on pancreatic ductal adenocarcinoma organoids. Two analyses were used to assess the effect of drugs, which included measuring the cell death ratio and calculating the area under the curve [118]. An interesting study by Sharick et al. used optical metabolic imaging to assess the metabolic response of breast and pancreatic tumor patient-derived organoids in drug screening [119].

Different tumor organoids have distinctive drug susceptibility features. This fact may indicate genomic changes and allow for detecting gene associations with therapeutic drugs. This provides high-throughput drug screening [50]. Verissimo et al. demonstrated that the presence of mutant RAS in colorectal tumor organoids strongly correlated with resistance to targeted therapies [120]. Another example was that AR (androgen receptor)-amplified prostate tumor organoids were sensitive to the inhibitor enzalutamide. AR-negative prostate cancer organoids reacted in the opposite way to this drug. In addition, prostate cancer organoids that carried a loss of PTEN function and a mutation in PIK3R1 were susceptible to everolinus [121]. Organoids obtained from the patient’s ovarian cancer tissue have been tested with several drugs that are used to treat high-grade serous ovarian cancer. It has been shown that wild-type TP53 tumor organoids were sensitive to nutlin-3 compared to mutant tumor organoids [95]. In addition, ovarian cancer organoids with a mutation in BRCA1 were susceptible to platinum drugs, compared to organoids derived from clear cell ovarian cancer [122].

Currently, patents are being created for the development of new substances for tumor therapy and the study of various inhibitors of tumor cells using 3D tumor models. Patented substances include the ATP inhibitor (957.EP3094317), transforming growth factor (TGF)-β inhibitor (WO2020104648) and curaxin (US20150045406). In addition, an invention has been patented relating to the development of a cancer vaccine where tumor organoids can be used (US20190343939). 

The use of organoids for modeling and studying TME is essential. For example, the immune microenvironment of a tumor is modeled using organoids derived from patient tissue that retains the original spectrum of tumor T-cell receptors and successfully simulates immune checkpoint blockades. This study confirms that organoids can be used to study personalized immunotherapy [101].

The development of successful personalized antitumor therapies requires the sophistication of 3D screening models and the inclusion of TME agents that play a significant role in tumor progression. To establish a microenvironment as close as possible to a tumor, it is undoubtedly necessary to reproduce one of the main processes involved in the progression of oncology—the formation of new blood vessels (angiogenesis). It has been shown that this system can be reproduced in 3D models. For example, hepatocellular carcinoma cells cultured as spheroids with nonparenchymal cells express markers associated with neoangiogenesis: vascular endothelial growth factor (VEGF), VEGFR2, HIF-1α [123]. A prevascularized tumor model was presented by Ehsan et al. as a method for studying the initial stage of tumor progression. This model included endothelial tumor cells and fibrin matrix. In particular, these spheroids demonstrated vascular formation through the angiogenesis into the matrix and vascularization [124]. Wörsdörfer et al. showed that it is possible to form blood vessels with a hierarchical organization in organoids. In addition, it has been shown that these blood vessels can integrate with the host’s circulatory system. Thus, this model can be used for long-term modeling of diseases to study the influence of angiogenic factors in tumor progression [125].

Extracellular vesicles are one of the key mediators in TME. Vesicles play a role in intercellular communication due to their ability to transfer various molecular components (lipids, growth factors, microRNA, etc.) [126,127]. It has been shown that vesicles of human colon fibroblasts induce the colony formation of colorectal cancer organoid cell colonies under hypoxia [128]. Moreover, the presence of tumor vesicles in the tissues of patients can be considered as biomarkers of cancer. The identification of vesicle proteins in the medium after tumor organoids cultivation may become a new approach for the detection of biomarkers [75]. The investigation of biomarkers can also be carried out by comparing the transcriptional differences between the lines of healthy and tumor organoids [87].

TME consists of various cells. Special attention should be paid to CSCs, which play a significant role in the progression of the disease. Linkous et al. created a model of cerebral organoid glioma, which consists of patient-derived glioma stem cells and human cerebral organoids [129]. This model is characterized by an increased number of neural progenitor cells and corresponds to the primary parental tumor [130]. It was shown that tumor stem cells penetrate and proliferate within organoids, giving them resistance to therapeutic drugs [129]. Thus, this model demonstrated the possibility of studying the behavior of tumor stem cells, which are an important target in the treatment of oncological diseases. It should be noted that it is possible to create a 3D engineered glioblastoma tissue construct using a bioprinter. For example, Tang et al. developed a model of glioblastoma using this method. This model included patient-derived CSCs, macrophages, astrocytes and neural stem cells. These cells were embedded in a hydrogel with a high hyaluronic acid content. The authors argue that this model allows for expansion of the possibilities of studying the tumor in comparison with spherical models. The 3D engineered glioblastoma tissue made it possible to study the effect of immune cells in the development of glioblastoma. In addition, it displays the transcriptional signature of the tumor and allows to measure the therapeutic efficacy of drugs [131]. Thus, certain additions to TME 3D tumor constructs may contribute to the improvement and consideration of these models as more reliable preclinical models resembling in vivo cancer phenotypes.

Another promising direction is the organoid biobanks creation. Biological material for further screening of new therapeutic agents at the stage of preclinical development can be stored in biobanks. For example, Sachs et al. created a living biobank organoids of breast cancer [47]. Ranges of investigations have been performed, including histological studies, DNA and RNA sequencing. Breast cancer organoids reproduce a diverse genomic tumor landscape, including copy number alterations and gene mutations. Differences in the expression of cell adhesion genes were also found. CRISPR/Cas9-mediated gene editing has been used to manipulate breast organoids and create functional TP53 mutants [38].

Nanki et al. created a biobank consisting of genetically engineered stomach organoids carrying various tumor mutations to study the genetic events of transforming cells [94]. Vlachogiannis et al. created a biobank of organoids from gastrointestinal tumors [132]. Phenotypic and genotypic profiling of organoids showed a high degree of similarity with the patient’s native tumors. Additionally, Tiriac et al. created a biobank of pancreatic tumor organoids and showed that these organoids exhibit heterogeneous responses to standard chemotherapy [133]. Bruun et al. created a living biobank of organoids from colorectal cancer liver metastases to study inter-patient variation in drug sensitivity and create a platform for ex vivo pharmacogenomics profiling. The authors used 39 tissue metastases from 22 patients. After analysis, it was possible to predict the most appropriate drug and assess the sensitivity to the drug; for example, organoids of patients 25 and 29 were sensitive to drugs in phase 2/3 clinical trials [134].

Pauli et al. developed a precision platform for the treatment of oncological diseases with 56 tumor organoid cultures and 19 xenograft models obtained from patients created [4]. Organoids and xenograft models have been used to conduct drug screening targeting mutated pathways. These pathways have been identified using whole exome sequencing. This research confirms the possibility of comparing the effectiveness of specific drugs on organoids, providing recommendations for patient care on an individual basis. It also allows determination of the next therapeutic steps for cases where standard clinical options have already been exhausted [4]. Thus, these data highlight the usefulness of the biobank for screening new therapeutic drugs, bypassing additional costs and time constraints, prior to in vivo trials.

Another feature of organoids is that they can be implanted into immunodeficient mice. This allows researchers to study the patterns of tumor growth in vivo and determine their sensitivity to drugs [135,136]. Animal drug testing is an integral part of preclinical research. The creation of a personalized model that includes an organoid and a recipient animal will provide an accurate mechanism for testing the efficacy and potential toxicity of new drug combinations and will ensure the identification of drugs targeting tumor-stromal interactions. Pauli et al. demonstrated the possibility of using this model, where the drugs’ effects (afatinib, vorinostat, paclitaxel, carboplatin, etc.) were tested, Kopper et al. also confirmed the possibility of testing the sensitivity of organoids to drugs after their subcutaneous injection in mice and Lee et al. investigated therapeutic responses to treatment of bladder tumors [4,137,138]. This model will provide new preclinical data for appropriate cancer therapy selection.

## 5. Conclusions

Recently, the search for an effective in vitro screening system for anticancer agents has been one of the most important tasks in medicine. The development of 3D tumor models can significantly increase the efficiency of screening for therapeutic drugs. Moreover, the next generation of anticancer drugs should focus on the malignant phenotype (growth, migration, invasion and angiogenesis of a tumor), not just cell proliferation. The 3D tumor models can provide a unique platform for studying important cancer events not available through other models. For example, xenograft models are closer to native tissue, but they are expensive, laborious and do not lend to high-throughput drug screening. It is difficult to develop xenograft models for slowly growing cancers. Another promising direction is the creation of the organ-on-a-chip model. This model allows recreating tissue–tissue interfaces and a dynamic physical microenvironment. However, the advantage of spheroids/organoids is the ability to get quick results and to increase the number of experimental replicas compared to the organ-on-chip model.

The use of 3D tumor models in personalized medicine, undoubtedly, remains a promising direction in the therapy of oncological diseases. The use of various approaches to study 3D patient models, such as genomic, metabolomic and proteomic approaches, will significantly improve the prediction of drug response and dosing regimens. The study of 3D model epigenetics will allow for the development of epigenetic change inhibitors and the creation of epigenetic drugs. It should be noted that the development of a personalized screening system is just beginning to grow, and for its successful implementation in practice, there are still many problems to be solved and drawbacks to be eliminated. The problems include the impossibility of expanding some lines of organoids for a long time, which can be eliminated by improving the nutrient medium; growth factors or low molecular weight inhibitors in the culture medium can have a significant effect on gene expression and signaling pathways in organoids, which can affect drug sensitivity. Perhaps, in order to obtain reliable results, it is advisable to combine various methods and model systems for the study of a tumor, which will improve the prediction and predictability of screening anticancer drugs systems.
